# No Place to Hide? Regional Resilience and Vulnerability to Global Catastrophic Risk

**DOI:** 10.1002/gch2.70146

**Published:** 2026-08-23

**Authors:** Florian Ulrich Jehn, Maximilian Rössler, Luke Kemp, Michael Cassidy, Zachary Kallenborn, Juan Bartolomé García Martínez, Lara Mani, Matt Boyd

**Affiliations:** ^1^ Center For Critical Computational Studies (C^3^S) Goethe University Frankfurt Frankfurt am Main Germany; ^2^ Senckenberg Research Institute and Natural History Museum Frankfurt am Main Germany; ^3^ Alliance to Feed the Earth in Disasters (ALLFED) Lafayette Colorado USA; ^4^ Societal Dynamics (SoDy) Toronto Canada; ^5^ Institute For International Legal Relations FernUniversität in Hagen Hagen Germany; ^6^ Centre For the Study of Existential Risk (CSER) University of Cambridge Cambridge UK; ^7^ School of Geography, Earth and Environmental Science University of Birmingham Birmingham UK; ^8^ Department of Geography King's College London UK; ^9^ Emerging Threats Group Oxford University Oxford UK; ^10^ Center For Strategic and International Studies Washington DC USA; ^11^ George Mason University Arlington Virginia USA; ^12^ Adapt Research & Islands for the Future of Humanity (IFH) Reefton New Zealand

**Keywords:** catastrophic failure, decentralization, democracy, food security, geomagnetic storm, nuclear warfare, pandemic, resilience factors, supervolcano, vulnerability

## Abstract

What places on Earth are most resilient to global catastrophic risk (GCR)? We provide the first study of what locations are more resilient against the impacts of nuclear war, near‐Earth objects, large‐magnitude volcanic eruptions, large‐scale cyberattacks, high altitude electromagnetic pulse, geomagnetic storms and pandemics. This shows there is no place on Earth which is resilient against all kinds of GCR. Australia shows resilience across the widest range of GCR scenarios in the literature we reviewed, but even for it, continued international cooperation and trade are essential. Across the different risks, common resilience factors that show up most are geographic isolation (e.g. islands), self‐sufficiency (especially in food production), high governance quality (more democratic and lower inequality) and decentralization to mitigate single point catastrophic failures (e.g. impacting trade or food supply). Many of these factors stand in tension with each other and trade‐offs are required to balance between different GCR scenarios and between a higher resilience against the immediate impacts or against the longer‐term consequences. The literature suggests that increased GCR resilience requires more investment in preparation (e.g., food security), planning (e.g., national risk assessments), and international agreements that facilitate cooperation on preparation and GCR response.

## Graphical abstract

No location on Earth withstands every global catastrophe. This review maps the geographic, institutional and infrastructural factors shaping resilience to nuclear, volcanic and impact winters, infrastructure loss and pandemics. Geographic isolation, food self‐sufficiency, strong governance and decentralization improve resilience across scenarios. Australia fares best, but remains vulnerable without sustained international cooperation and trade.

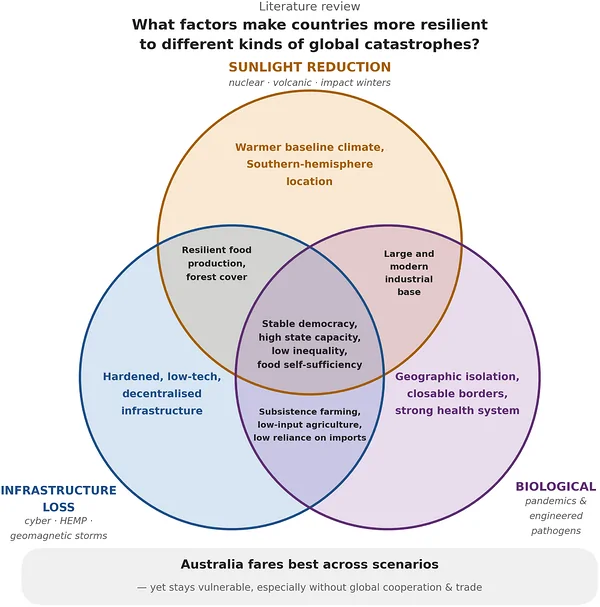

## 1 Introduction

Global catastrophic risk (GCR) has been defined as the risk of death of a significant fraction of humans or a significant loss of well‐being on a global scale. Most commonly this refers to death of at least 10% of the global population within months to years [1, 2]. Research around this topic has grown considerably over the past decade [2], with much of it being focused on hazards, namely events or processes that have the potential to inflict catastrophic harm upon humanity. But risk also consists of vulnerability and exposure to the hazard. Vulnerability is the characteristics and circumstances which make a system susceptible to damage, and exposure is that the system is exposed to the hazard [3, 4, 5]; United Nations General [6]. Others also add ‘response’ to this risk equation: how a system responds can alleviate or aggravate the risk [7, 8, 9].

Another important concept in relation to risk is resilience. Resilience is often understood as the ability to withstand shock and recover without a system losing its fundamental identity or function [10, 11, 12]. Specifically, we take resilience, in the context of global catastrophic risks, to be a society's capacity to maintain critical functions and human welfare over time when faced with severe disruptions. We are most interested in three interconnected aspects: (1) robustness—the ability to withstand initial shocks and minimize immediate harm; (2) recovery—the capacity to restore essential functions within meaningful timeframes; and (3) adaptation—the ability to learn and reorganize while preserving core capabilities, even if original structures or processes must transform. We can measure the degree of resilience by tracking how much welfare and functionality a system sustains over a specified timeframe following a catastrophic event. This is evaluated relative to either the system's pre‐event baseline or minimum requirements for human survival. A region's overall position with respect to GCR depends on both its exposure to specific hazards and its capacity to cope. This paper considers both.

What specific geographical, institutional, and infrastructural factors have been empirically or theoretically identified as enhancing regional ability to withstand and recover from a variety of global catastrophic risk? No literature to date has directly answered this question. While there are many papers on resilience to GCR, particularly in food systems [13] and in adjacent areas (pandemic preparedness; Boyd et al. [14]), and some individually explored factors like the resilience of large islands (such as Australia and New Zealand) [14, 15, 16, 17], no research to date has comprehensively mapped these factors together. Our review does so by considering factors based on geography (e.g., location, climate, resource availability), institutions (e.g., governance, policies, social cohesion), infrastructure and combined factors (e.g., built systems, technology, supply chains).

**FIGURE 1:**
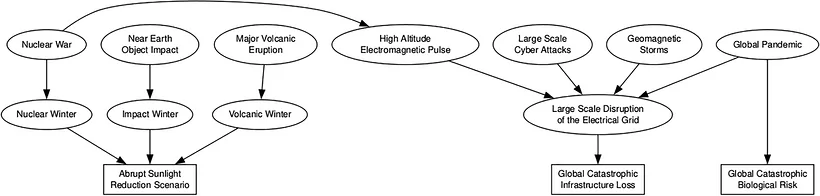
Overview of the different global catastrophic risks considered for this review and how they relate to each other.

Our review assesses three broad types of GCR: Abrupt Sunlight Reduction Scenarios (ASRS) [18], Global Catastrophic Infrastructure Loss (GCIL) [19, 20] and Global Catastrophic Biological Risk (GCBR) [21] (Figure 1). These were chosen since they are widely discussed in the GCR literature [13, 22, 23]. Each of these risks not only causes widespread direct deaths and destruction of infrastructure, but also significant cascading global consequences from these direct harms.

### 1.1 Abrupt Sunlight Reduction Scenarios

As the name suggests, ASRS are defined by an abrupt (meaning weeks to months) reduction in sunlight reaching the surface of the Earth. ASRS can be caused by soot blocking out sunlight due to firestorms caused by a nuclear war [24, 25], sulfate aerosols scattering sunlight after major volcanic eruptions [26, 27] and soot, dust and ash (and possibly sulfates as if the asteroid hits sulfur‐rich minerals; Rodiouchkina et al. [28]) blocking sunlight after asteroid/comet/meteor (bolide) impacts [29, 30]. Reduced sunlight leads to changes in the global average temperature, precipitation and wind speeds globally [24]. Likely consequences could be severe, including reduction in food production yields [31] and subsequent disruption of food trade [13, 32].

### 1.2 Global Catastrophic Infrastructure Loss

Risk from a GCIL is that infrastructure and industrial production are disrupted on a large scale (continental to global). As almost all infrastructure and industrial production is reliant on electricity, the most likely vector for a GCIL is disruption of the electrical grid for a prolonged period (a blackout). Besides the general disruption of all societal systems [33], this could also negatively impact food production due to a loss of storage, transportation and a decline in production due to loss of agricultural inputs like fertilizers [20, 34]. Such a disruption could hypothetically be caused by High Altitude Electromagnetic Pulses (HEMP) [35, 36], geomagnetic storms [37, 38, 39], globally coordinated cyber attacks [40] and pandemics so deadly that people stop showing up for work on a very large scale [41].

### 1.3 Global Catastrophic Biological Risk

Global catastrophic biological risk includes all biological causes for large biological hazards, both natural and man‐made. GCBR scenarios are characterized by widespread infectious disease causing mass casualties and severe societal disruption on a continental to global scale [21]. Such catastrophic biological events could arise from naturally emerging pandemics with high transmissibility and lethality, potentially far exceeding the impact of the Covid‐19 pandemic [42], accidental release of dangerous pathogens from laboratory settings [43], or deliberate deployment of engineered biological weapons [44]. Beyond direct mortality, which could number in the tens to hundreds of millions or more [45], these scenarios lead to cascading effects including the collapse of healthcare systems, loss of essential workforce capacity across critical sectors, breakdown of supply chains, and potential secondary famines due to disruption of agricultural production and food distribution. The combination of direct casualties and systemic collapse could result in mortality far exceeding the initial disease burden.

## 2 Methods

**FIGURE 2:**
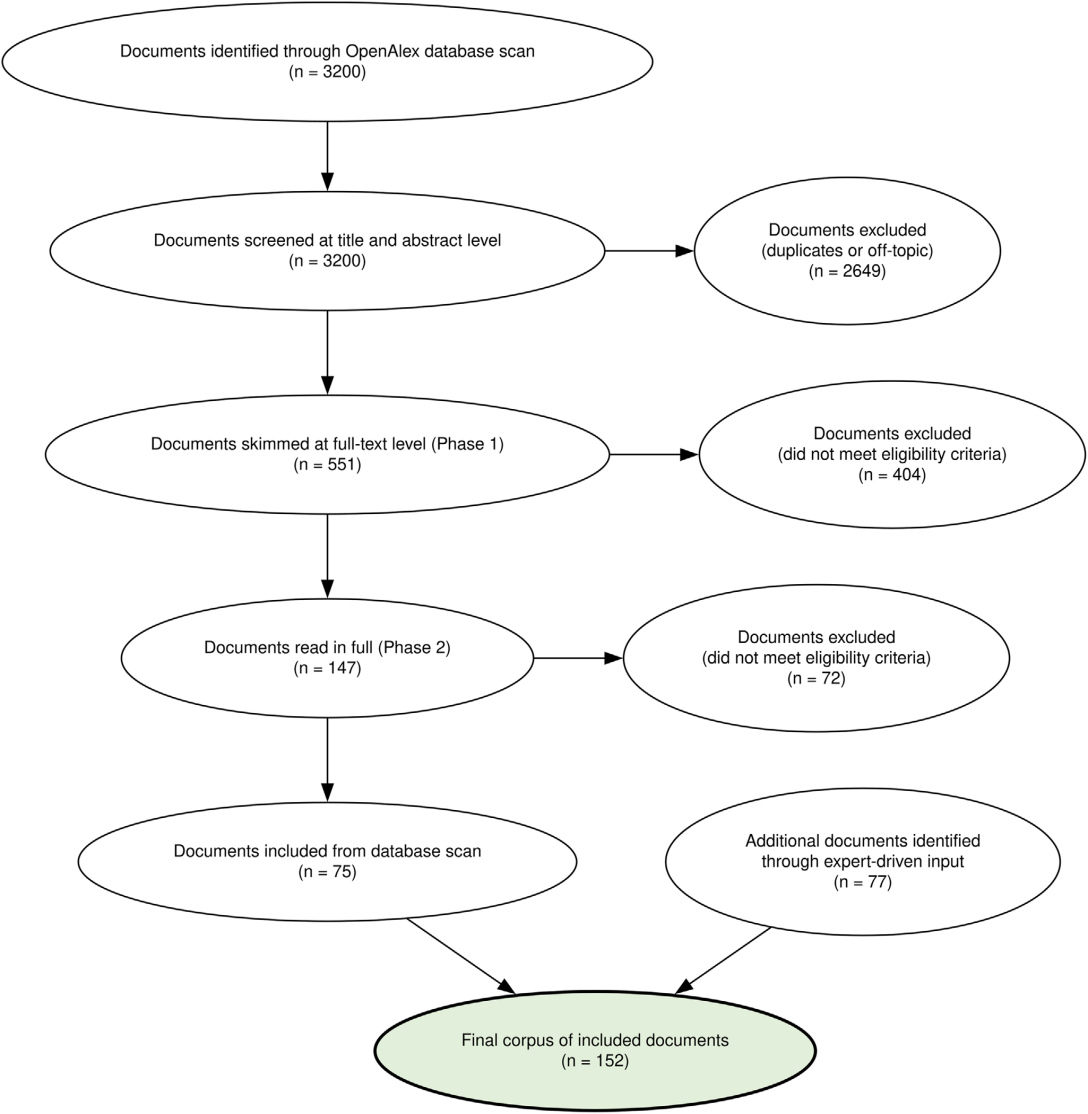
Selection process for documents used for this study.

This review combines two approaches: a structured database search and an expert‐driven curation phase. We adopted this hybrid design because the literature relevant to GCR resilience is highly heterogeneous in methodology, geographic scope, and disciplinary framing, and because much of it is not explicitly framed as GCR research and would therefore be missed by keyword‐based search alone. A flow diagram summarizing the selection process is provided in Figure 2.

### 2.1 Database Scan

The database for this study was a search using OpenAlex [46]. OpenAlex is a free and open source bibliographic catalogue, which can be queried for academic documents. The exact query we ran was (as of Oct 10, 2024): (resilience OR mitigation OR resilient OR mitigate) AND (((“global catastrophic risk” OR “existential risk”) AND (nuclear OR famine OR volcano OR pandemic)) OR (“nuclear winter” OR “volcanic winter” OR “abrupt sunlight reduction” OR “catastrophic infrastructure loss” OR “extreme space weather” OR “severe space weather” OR (“biological threat” AND pandemic) OR “global catastrophic biological risk” OR “engineered pathogen” OR “global food supply catastrophe”))

This query was meant to capture all documents which specifically tackle resilience from a GCR perspective and therefore we used terms commonly used in the literature like GCIL, ASRS or GCBR. It resulted in a total of 3200 matching documents. All relevant files can be found in the repository of the paper [47].

### 2.2 Eligibility Criteria

Documents were eligible for inclusion if they addressed at least one of the following: regional resilience to a GCR; regional variation in catastrophe impacts (including non‐GCR catastrophes where the mechanism described was potentially transferable to GCR contexts); cross‐regional comparison of responses to catastrophes; regional preparedness assessments relevant to GCR; analysis of local GCR resilience factors; or local assessments of resilience factors transferable to global‐scale risks.

Eligible documents also had to address one or more of the following GCR categories: ASRS (nuclear winter, volcanic winter, near‐Earth object impact), GCIL (geomagnetic storms, HEMP, cyber attacks, large‐scale infrastructure failure), GCBR (pandemics, biological weapons), or combinations and cascading interactions among these.

Documents were excluded if they addressed only: local disasters without global implications, regular natural disasters without GCR‐scale potential, or non‐catastrophic risks (e.g., individual mental health impacts of isolated events). Language was restricted to English. No restrictions were placed on publication type and date.

### 2.3 Selection Process

Title and abstract screening of the 3200 records was conducted by a single reviewer (MR), with random spot‐checks by FUJ and MB to verify exclusion decisions; all spot‐checked exclusions were confirmed as clearly off‐topic. At this stage, records were excluded if they were duplicates or did not mention resilience at any level relevant to GCR. This resulted in retention of 551 potentially relevant documents.

The 551 records were then skimmed at full‐text level by FUJ, MB and MR against the eligibility criteria above. Each document was assessed by all three reviewers. All documents were included where at least one reviewer deemed them relevant. This reduced the corpus to 147 documents.

### 2.4 Data Charting and Synthesis

At the outset, we developed a structured charting framework intended to extract for each document: GCRs addressed, geographic scope, whether resilience factors were already implemented or proposed, type of resilience factor (geographic, institutional, infrastructural, other), temporal focus (robustness versus recovery), methodological approach (model, review, case study), and nature of findings (quantitative, qualitative, or theoretical). In practice, this framework proved unwieldy given the heterogeneity of the included literature, and we moved to narrative synthesis informed by the framework rather than formal charting. The charting dimensions instead served as a shared frame of reference for what reviewers attended to when reading.

Claude Sonnet 3.7 was used to pre‐sort the 147 eligible documents by GCR category and geographic focus to allocate reading workload across the three reviewers. The model had no role in eligibility decisions, data extraction, or synthesis; all 147 documents were read in full by a human reviewer. This further excluded 72 documents as they did not meet the eligibility criteria when studied more closely, leaving 75 documents. Based on those 75 documents, each author wrote a synthesis for their assigned categories.

Resilience factors were identified inductively. Each reviewer recorded any factor that a source paper explicitly linked to resilience against one or more GCRs, independent of whether the supporting evidence was empirical, modelled, or theoretical. A single mention was enough to be included, since much of the GCR resilience literature is still small and exploratory. Where source papers reported conflicting findings (e.g., the contested magnitude of UV exposure following nuclear war, or the disputed severity of geomagnetic storm impacts), both positions are reported.

The three reviewer‐specific syntheses were then merged by FUJ, who wrote a synthesis, which was then re‐ordered by GCR category level, that is, the identified factors relevant for (Level 1: All GCR, Level 2: ASRS, GCIL, GCBR, Level 3: Nuclear winter, volcanic winter, impact winter, geomagnetic storms, HEMP, cyber attacks, pandemics). This allowed us to assess the resilience factors by catastrophe type, cross‐cutting themes and identify regions which are likely most resilient.

The category structure used in Section 3 (governance, production, location, trade, and additional factors at the general level, followed by hazard‐specific subsections) emerged inductively during the merging stage rather than being pre‐specified. After FUJ consolidated the three reviewer syntheses, recurring themes were grouped under category headings, and the same exercise was repeated within each GCR subcategory. Factors appearing in syntheses across multiple GCRs were assigned to Section 3.1; factors specific to one GCR were assigned to the relevant subsection.

Country‐level identifications in Section 3 follow a two‐step logic. First, we built a ledger (which is available in the repository of this paper; Roessler and Jehn [47]). This ledger contains each named mention of countries we extracted from the documents (and the source that mentions them), as well as all inferences we made on which countries are likely resilient based on the resilience factors from the literature we used. Second, we counted up how often different countries came up in both the named and inferred mentions. This allowed us to compare which countries tend to have the most resilience factors described in the literature.

Where countries appear in the synthesis but are not named in any source paper (e.g., Switzerland and Uruguay in the GCIL section), the identification is an inference by the authors applying the factor list to known country characteristics. Such inferences are not exhaustive: other countries likely fit the factor list comparably well. The country‐level conclusions should therefore be read as illustrative of the kinds of places favored by the identified factors, not as a definitive ranking. A more systematic country‐level analysis would require a composite index built from quantitative indicators for each factor, a direction we discuss in Section 5.3.

### 2.5 Expert Driven Input

The database scan provided a broad overview of the literature, but also highlighted that there were important gaps, as much of the relevant literature is not explicitly framed in the context of GCR. For example, there is a wide range of volcanic research that is highly relevant, but was not included. Therefore, we invited additional authors with subject matter expertise around societal collapse (Luke Kemp), nuclear winter and food systems (Juan B. García Martínez), cyber attacks (Zachary Kallenborn) and volcanic eruptions (Lara Mani, Michael Cassidy) to contribute to the review and fill as many of the remaining gaps as possible. All authors contributed further studies across all sections of the manuscript.

This expert‐driven phase was deliberately unsystematic: each contributing expert identified additional literature based on their domain knowledge, without a shared search protocol. We chose this approach because the gaps we sought to fill could not be reliably identified through keyword‐based search and had to rely on expert judgment. Experts were briefed on the scope and aim of the paper and asked to add documents that would contribute to these aims, but had been missed. A total of 77 additional documents were added through this process, bringing the final corpus to 152.

## 3 Which Factors Make Places Resilient to GCR?

The combination of the structured database search and the expert driven input allowed us to sample a wide range of the literature relevant to GCR resilience. In the following, we describe what makes societies resilient to different types of GCR based on this literature. Some of the factors that influence resilience to GCR are shared between different hazards. Therefore, for this section we will start with those more general factors and then examine the specific hazards.

### 3.1 Factors Applicable Across All Global Catastrophic Risks

There are several factors that make a society more resilient to all GCR. These mostly group around the general capabilities and stability of a society. If a society is more politically stable and able to produce a wide range of goods, the literature suggests that this society should be more resilient.

#### 3.1.1 Governance and Institutional Factors

The literature repeatedly identified effective governance as a critical resilience factor, as all else is downstream from that. If a society does not have governance structures that work, it will not be able to efficiently mobilize or distribute capabilities and resources and react quickly, even if it has other resilience factors. Effective governance encompasses social cohesion [48, 49, 50], political stability, defense capability, education levels, health security systems, governance structures, catastrophe planning, risk communication, and social capital [15]. Similarly, for handling hazards, having a clear hierarchy between levels of government makes it easier to coordinate [33]. Also, higher state capacity allows a better implementation of measures to handle hazards [51, 52, 53]. Relevant planning domains identified include national emergency management, agricultural coordination and food resilience planning, and fuel storage or biofuel production policies [54], alongside public awareness of resilient food systems [55].

Democratic processes were also identified as hallmarks of resilient societies. Democracy enables more flexible reactions, by enabling epistemic humility to interpret events, collective intelligence mechanisms (democratic processes, prediction markets, diverse decision‐making), and good data‐sharing for adaptive responses [56, 57]. Boyd and Wilson [58] argued that this could be further enhanced by the implementation of dedicated GCR policy structures, such as parliamentary commissioners for extreme risk, or similar institutions. Also, decentralized decision‐making (historically associated with greater innovation than centralized systems), like differences in the history of agriculture innovation in decentralized Europe in comparison with more centralized systems [59, 60] and international cooperation for globally‐scaled risks have been identified as increasing resilience [61]. We identified some historical evidence to support the importance of democratic processes to be better able to handle GCR. Historical analysis using the Late Antique Little Ice Age (ca. 536–556 CE) as a nuclear winter analogue demonstrates better outcomes correlate with broad political participation, bridging social capital through broader stakeholder engagement, decentralized decision‐making, shared authority structures, and horizontal information flow [22, 49, 50]. Similarly during Covid‐19, more democratic island nations implemented more effective border biosecurity and exclusion/elimination strategies resulting in fewer pandemic deaths, possibly due to social cohesion, perceived legitimacy, and feasibility of interventions [62].

The included literature suggests that social resilience may be more influential than environmental resilience in determining outcomes [61, 63]. Other potentially important social factors included trust in government and social cohesion, low inequality, food storage flexibility, as well as agricultural diversification. Historical case studies show the importance of these factors: for example, during the Justinian plague, political adaptation and absence of infighting (social cohesion) contributed to recovery [64], while low government corruption, trust in government, and trust in individuals were key predictors of early Covid‐19 pandemic outcomes [65]. High inequality has been identified as a historical vulnerability factor in large dataset historical studies [22, 66], as well as been highlighted as a risk for future generations [67] and as an important contributing factor to GCR in general by decreasing social cohesion and weakening democracies [68]. During Covid‐19, non‐island jurisdictions with lower income inequality suffered less excess mortality and less severe initial GDP contractions [62]. Higher inequality also appears to drive higher corruption, polarization, and democratic backsliding. Importantly, it also seems to be increasing for the majority of the global population [69].

#### 3.1.2 Production

A second theme identified in the literature we assessed is a society's productive capacity, especially its industrial base. The more capacity you have, the more of a buffer exists. For example, a larger industrial base means more things like paper mills, biorefineries and breweries, which could be repurposed for food production [70, 71] or food oil refineries, which could produce biodiesel [54]. More generally, there will be more institutional capacity for rapid budget redirection and industrial resource reallocation toward resilient food production [19, 72]. Having a larger productive capacity also implies more resources, which could be used to create strategic stockpiles of fuels, seeds, fertilizers, and other critical inputs [15], but it is unclear how different societies compare here. Finally, this larger productive base could also be repurposed to create manufacturing capability for replacement parts if these are not available anymore via trade [15]. A historical precedent here is the volcano climate shock after the Tambora eruption, where famine in Europe could only be averted by imports from Russia [73].

Not all industrial capacity is directly translatable to be helpful after global catastrophe, but generally the larger the industrial base is, the higher the chance that GCR relevant industries are also present. Some industrial capacity can be repurposed relatively quickly, like the transition from cars to fighter planes in the US in World War 2 [74] and the shift to produce much more disinfectant during COVID‐19 [75]. Industrial production will have inherent sectorial differences that limit transfer, such as the nature of technology (semiconductor fabs cannot produce vaccines), the global concentration of specific industries, and the degree to which crucial supply chains are globalized. Nonetheless, the overall size of the industrial base can be a useful proxy for general resilience.

#### 3.1.3 Location

Large islands were often identified as good places for resilience to GCR, as they are climate buffered due to the heat capacity of the water around them, and can easily isolate themselves (e.g. via closed borders), as it is more difficult to reach a location when there is a large water body in between [16, 76]. Generally, relatively isolated regions with self‐sufficient food production could also prove to be very resilient, with the important part being their remoteness; islands tend to be more remote [77]. However, they have to have an economy that is able to at least support itself partially. Especially fuel and medication can quickly become a problem if no infrastructure exists on the specific island to produce those goods [54, 78]. Having a large soil seed bank could be an advantage, as it allows to potentially create new cultivars that are better suited to changed environments and allows plants to regrow easier [79]. Also, once catastrophe strikes, urban areas quickly become death traps, as they are highly reliant on continuous import of foods and other daily products [33, 77]. We specify this resilience factor to ‘large islands’ (such as New Zealand, but also Australia, as it is a continent sized island) since small islands (such as Tuvalu or the Marshall Islands) are significantly vulnerable to sea‐level rise from climate change, tsunamis from earthquakes and near‐Earth object impacts, and tend not to have the economic size to be self‐sufficient. However, we also note that some islands could maintain traditional ways of life, even after global catastrophe, as they have ample food production and are significant above sea level (e.g., Vanuatu and Solomon Islands). Many of the problems described here only apply to regions that are overpopulated relative to their local carrying capacity and reliant on a steady inflow of trade.

Location also determines the exposure of a country to different hazards. For instance, countries in the southern hemisphere will suffer less of a drop in temperatures during a nuclear winter [24], which is explained in more detail below. Similarly, countries in the tropics, Gulf region, and Subsaharan Africa are far more exposed to climate impacts, especially extreme heat [80, 81].

#### 3.1.4 Trade

The papers we assessed implied that societies particularly reliant on trade to receive needed goods like fertilizers, food, or medicine may be more vulnerable to global catastrophic risk [13, 15]. Catastrophes that are not overtly global in scope, such as low to moderate magnitude volcanic eruptions or wars blocking important trade routes (e.g., Strait of Hormuz), could still cripple global trade and communications if affecting key ‘pinch points’—regions where a high convergence of global systems and infrastructures are observed [82]. Critical trade dependence might be partly mitigated by ensuring robust regional trade networks [83]. Resilience may require food or fuel rationing and prioritization systems, proactive inventory management, and open trade policies. Export bans may be particularly harmful as there is potential for cascading impacts across many trading partners. Trade policy coordination, especially to manage hoarding behaviors, requires proactive management of food supply systems and transportation networks [13, 84, 85].

#### 3.1.5 Additional Reasons

More wealthy countries tend to have more resources and thus capabilities than poorer countries. This is acknowledged in the Global Catastrophic Risk Index by the Global Governance Forum [86], as it tracks the resilience of countries against GCR based on proxies like economic stability, education or business environment which are all strongly coupled to how rich a country is. A real world test of the effects of wealth was the COVID‐19 pandemic, which showed that richer countries tended to navigate the pandemic better, but also that wealth only captures a part of the resilience [14]. In addition, the expectation is that food prices will rise considerably in many of the scenarios described here and thus richer countries can afford more food [87, 88].

Especially for the food system, resilience factors that get regularly mentioned are modularity, decentralization and diversity (e.g., in the form of genetic diversity in crops) [89, 90].

#### 3.1.6 Summary of General GCR Resilience Factors

Taken together, societies are generally more resilient to GCR if they:

- Are more democratic.
- Are wealthier.
- Have lower inequality.
- Have large industrial base, with high governmental influence.
- Are located in remote locations (like large islands).
- Have higher decentralization and diversity.
- Are not highly reliant on trade or have geographically close trading partners.

There are tensions here, especially between being an island and having a large productive capacity. Relaxing this a bit, Japan could arguably fit these factors reasonably well. It has a large industrial base, with high governmental influence and is located on an island. It is are also democratic and have relatively low inequality. However, both are very reliant on trade. If we relax the factors, this could further include Australia, New Zealand, the United Kingdom and Iceland.

### 3.2 Abrupt Sunlight Reduction Scenarios

Southern Hemisphere islands may be particularly resilient against ASRS, because there are no nuclear armed states in the Southern Hemisphere and also fewer large volcanoes. In larger ASRS, although effects occur in both hemispheres, the hemisphere in which the triggering event occurs tends to suffer more sunlight reduction [24]. In addition, oceans around islands act as a temperature buffer, which allows them to maintain a more stable temperature. Boyd and Wilson [15] argue that Australia, New Zealand and Iceland have the best prospects among islands in an ASRS, as they possess diverse resilience factors including enough capacity to produce food to maintain their population. Other evaluated islands like Vanuatu or the Solomon Islands produce large food excess but lack infrastructure and manufacturing to sustain industrial society without trade, while island societies like Indonesia may be at risk from social or political instability during an ASRS.

In an ASRS food production could likely be affected on a large scale and in more severe scenarios most countries do not have suitable cultivars available in their borders. However, regions with diverse climate zones and good landrace crop varieties have at least a higher chance of having marginally suitable cultivars to handle the colder temperature in ASRS [91]. As the growing season could be shortened during ASRS, countries with a long (or full year) growing season could fare better, as they have a longer buffer [92]. In addition, societies with larger food production sectors and diverse crops could fare better. Examples highlighted in the literature are Brazil and Argentina [85]. To supplement local food production, trade would likely be necessary for many countries. Countries with a network of trading partners throughout the world and with many climate regions could be in an advantageous position [93].

High reliance on fisheries is seen as a detriment during ASRS. While there might be some areas in the tropics and subtropics with increased catch, generally catch is predicted to decrease during ASRS [94]. This pattern was also seen during the last major ASRS after the eruption of Mount Tambora (Indonesia, 1815), which led to much reduced catch in many regions [95]. This could likely be repeated in a new ASRS. Seaweed, in contrast, has been identified as having a high chance of being able to contribute a significant amount of global calories during ASRS, especially in the Pacific area (South East Asia and Western side of South and Central America and in some seas near major river deltas like Niger or Congo) [96].

When it comes to energy, researchers have found that areas that have more renewable energy could fare worse, as both wind and solar decrease during ASRSs [97], but fossil fuel reliance could also make energy production difficult, if trade ceases [54]. This could be mitigated in areas with either easy access to natural energy sources like geothermal or hydropower or large amounts of wood available for fuel [98]. Heavily forested regions could also be used as a source of wood‐based foods like Fungi [99]. Areas without wood, but a large industrial base, could produce advanced resilient foods for low sunlight scenarios like methane‐derived single‐cell protein, which demand specific facilities for microbial biomass cultivation [100, 101, 102], locations with existing chemical or bioprocess industries possess advantages for rapid deployment of synthetic food production systems [72]. Success in implementing such a repurposing requires organizational capability, pre‐existing engineering designs and fast‐build methods [85].

A factor just recently identified in the literature is the danger to the drinking water system. Lamilla Cuellar et al. [103] analyzed changes in frost depth during a nuclear winter and how they could impact drinking water. They found that most drinking water infrastructure further north than 25° latitude could be damaged. This means from a drinking water perspective being in the subtropics, tropics or Southern Hemisphere is preferable, though there are mitigation measures [104].

Finally, Maher and Baum [61] identified equatorial mountains as a good place for food stockpiles during ASRS. They have physical protection due to elevation, are less affected by sunlight reduction and are far away from possible direct impacts like tsunamis. The equatorial Andes (e.g., La Paz, Bolivia; Pasto, Colombia) are specifically mentioned as prime candidates for food stockpile placement.

#### 3.2.1 Nuclear Winter

**FIGURE 3:**
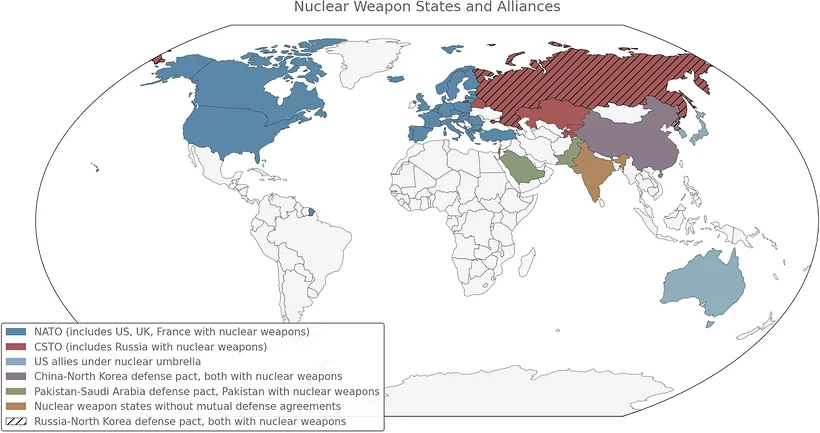
Nuclear weapon states and their military alliances.

The direct effects of nuclear weapons harm those areas targeted during any nuclear conflict. Targeted locations, cities, and regions would suffer immensely and the top of the list of targets would be those societies involved in nuclear war, likely to be societies which also possess nuclear weapons. This means the United States, Russia, United Kingdom, France, China, India, Pakistan, Israel and North Korea are in most danger, as are countries that are in conflict with one of those powers (e.g., Ukraine being at risk from Russia, Iran being at risk from Israel or the United States). This risk further increases for conflicts where both parties have nuclear weapons. Also, for any conflict that would include the United States, United Kingdom or France, there might be a high chance that NATO could get involved (collective defense after Article 5), which means that NATO countries are generally also more in danger from nuclear war. Additionally, countries under a nuclear umbrella (like Japan or Australia) might be at elevated risk (Figure 3). Non‐NATO and especially non‐target societies could likely suffer less direct harm [24, 31]. In particular, many remote islands are unlikely to be direct targets [15]. This all points to the Northern Hemisphere as being highly vulnerable to nuclear war, while the Southern Hemisphere might fare better.

Climate disturbances are likely to follow the same pattern, with high latitude regions, especially in the Northern Hemisphere, suffering the most cooling (with corresponding shortening of growing season, and mid‐latitude regions experiencing the highest UV exposures if the nuclear war has disrupted the ozone layer) [24, 92]. Potentially elevated UV exposure could occur especially in the tropics [24, 105]. The intensity of the UV exposure is still a topic of active debate. With some estimates stating that UV exposure would only be a problem in the largest scale nuclear conflicts and only years after the war [106], others argue that even smaller nuclear war scenarios could lead to strong disruption of the ozone layer and thus UV exposure [107].

Specific locations described in the papers examined as being more resilient to the climate and food system impacts of nuclear winter include: Australia [15, 17, 92, 96], New Zealand [15, 17, 54, 92, 96], Iceland [15, 54], Solomon Islands or Vanuatu [15], Peru [94, 96], Chile [96], the Southern Ocean [24], Pacific Ocean equatorial upwelling zones [96], and equatorial regions generally [91], as well as Southern Africa and South America [92].

Specific countries and regions identified as being particularly at risk include: high latitude regions [24, 31], low population countries with insufficient agricultural production and food stores, non‐tropical countries that cannot grow crops during nuclear winter [85], the United States [24, 92, 94, 96, 108], Central Europe [13, 96], Eastern Europe [24, 96], Russia [24, 84, 94, 109], Canada [84, 94, 96], China [24], North Korea [84], Asian monsoon regions [24], Israel, Iran, and Pakistan [109], and Mediterranean climate zones [79].

#### 3.2.2 Volcanic Eruptions

**FIGURE 4:**
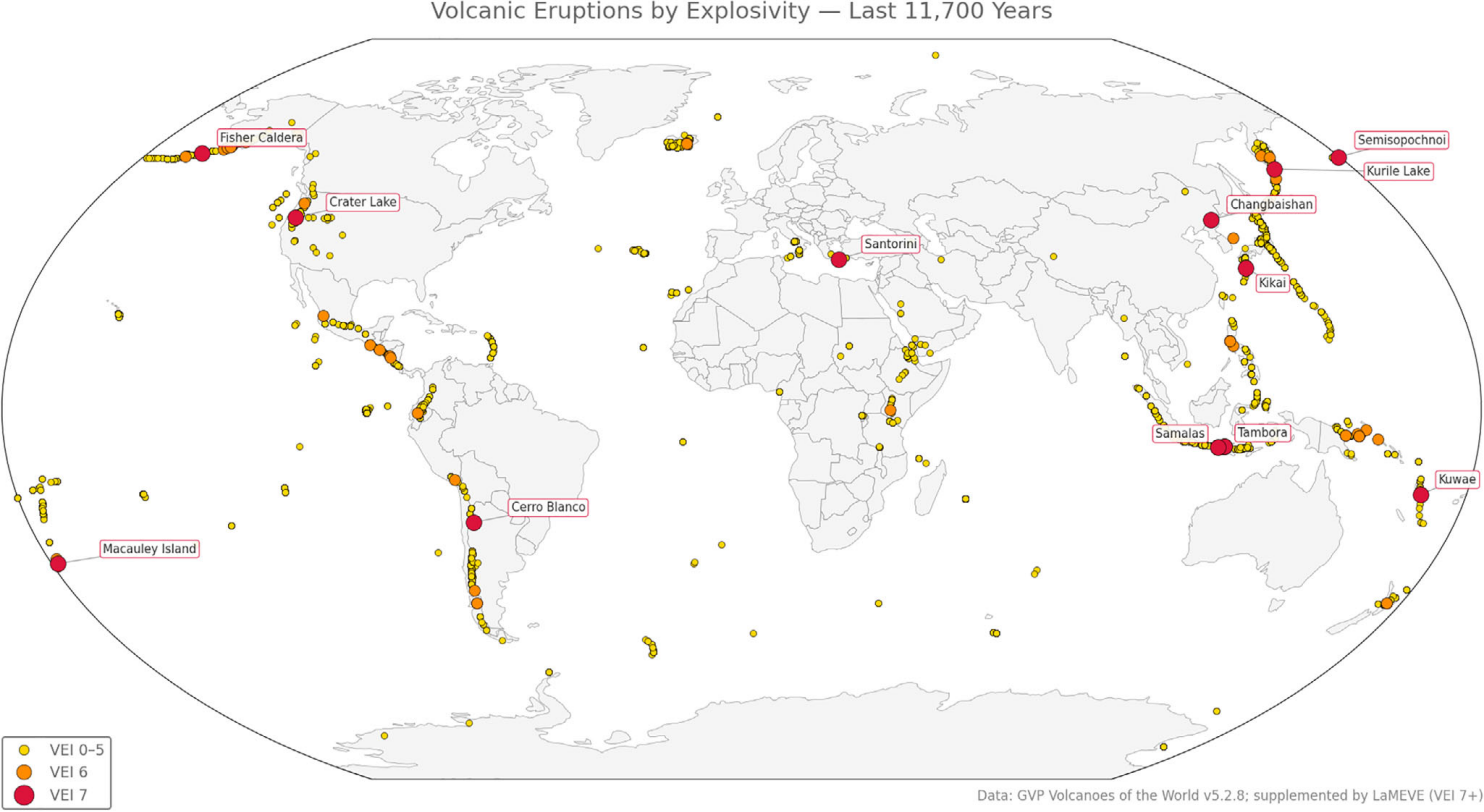
Global distribution of known volcanic eruptions in the Holocene (last 11 700 years) for VEI 0–7. Note due to incomplete volcanic records, especially further back in time, this map does not capture all volcanic eruptions in the Holocene. Colored by Volcanic Explosivity Index (VEI). Based on Global Volcanism Program and Venzke [112] and Crosweller et al. [113]. VEI 7 Holocene eruptions are labelled.

In terms of direct impacts (non‐climatic impacts) from volcanic eruptions, for instance ash fallout, hazardous flows (pyroclastic, lahar, lava, mass movement and accompanying tsunamis), the volume of explosively erupted magma is a major determinant on the extent of global catastrophic impacts. These direct impacts are most relevant for volcanoes that have the potential for eruptive volumes of Volcano Explosivity Index (VEI) 7 or 8, with VEI 8 (with volumes >1000 km^3^) representing ‘super‐eruptions’. Ash from such eruptions could blanket entire continents. For example, even the VEI 7 eruption of Tambora led to ash fall in a large part of South‐East Asia [110]. This affects food, water, energy and financial security. Ash deposition on land, if large enough, could have a climatic impact due to increases in surface albedo [111]. The geographic location of these large eruptions is equally important (Figure 4).

Take the example of the Hunga Tonga eruption in 2022, the highest intensity eruption recorded with modern instruments. A submarine eruption, much of the ash (and gas) distribution occurred in the ocean, triggered a shockwave with an accompanying tsunami, an eruptive plume reaching 55 km high and underwater flows that severed the international and domestic communication cables to the Kingdom of Tonga [114, 115, 116, 117]. Despite its scale, the eruption's global impact was far less than it could have been due to its location in the middle of the pacific, >60 km from the nearest populated islands and away from globally critical infrastructure [118]. Mani et al. [82] highlight the regions where clusters of critical infrastructure (or trade routes) lie in proximity of volcanic regions, these so called ‘Pinch Points’ are especially vulnerable to volcanic activity, due to the effects on global cascading risks.

The regions most in danger are South East Asia, the Mediterranean and Pacific Northwest. In terms of population exposure to volcanic risks, regions like South East Asia, Central and South America, Southern Europe, and parts of Africa (along the East African rift valley) have the most amount of people living within close proximity of active systems [119, 120, 121]. The vulnerability and resilience for these regions posed by large eruptions is largely unstudied, however Latin American volcanoes have been ranked against these metrics to understand regional risk (e.g., Reyes‐Hardy et al. [122]). As the majority of global volcanoes go unmonitored [123] (especially more acute in poorly resourced countries), and that more than 90% of eruptions larger than VEI 6 have periods of more than a century between eruptions, it seems likely that few countries will have the sufficient warning, preparedness and learned resilience to cope well with large eruptions in their own borders or neighboring regions. Regions that are better resourced in the area include, US, Japan, Iceland and New Zealand. Regions which have greater learned volcano resilience include Indonesia, Iceland, and the Philippines.

When assessing the indirect impacts from volcanic eruptions such as climatic impacts, the VEI (i.e., volume of the volcanic eruption) is less significant [124]. Instead, the amount of sulfur gas emitted, the eruption latitude, the season and whether there have been clusters of eruptions mainly control the magnitude of climate cooling and longevity or response. Cooling shock following sulphur aerosol forcing is one factor that influences global catastrophic risk, and climate models and proxy records (such as tree rings) point to greater impacts in the Northern Hemisphere, with islands and southern hemisphere buffered by the temperature capacity of the larger southern oceans. Just as consequential are the simultaneous extreme weather events that also accompany a volcano climate shock (including, droughts, floods, storms and frosts), and the disruptions it can inflict on major climate teleconnections such as the monsoon (particularly African, South and South East Asian monsoons) and the El Niño Southern Oscillation (ENSO). There is little data on the regional effects of supervolcanic eruptions and implications for GCR resilience. Following the Toba eruption (around 74 000 years ago) India and Sub‐Saharan Africa fared reasonably well as climate refuges [125].

#### 3.2.3 Near‐Earth objects

Generally near Earth object impacts lead to similar effects as other ASRS, but there seems to be no clear best place in general, because the impact location is randomized, depending only on which side of the Earth is in the path of the asteroid during impact. However, as much of Earth is covered by water, there is a high chance that an ocean could be hit, which could cause massive tsunamis. Though the size and thus impact of such tsunamis is a topic of ongoing debate [126]. Still, this means places further away from large water bodies could at least not have to face this additional hazard [127]. Countries with higher elevated ground will also be less exposed.

#### 3.2.4 Summary of Factors Relevant for ASRS

In addition to the general features identified in Section 3.1.6, ASRS resilience could be increased by the following factors:

- Southern Hemisphere location, as there are fewer volcanoes, no nuclear weapon states, less endangered drinking water systems, and the larger ocean area acting as a temperature buffer.
- Being a food self‐sufficient island.
- Societies with diverse climate zones and thus a more diversified agriculture.
- Societies with a longer growing season.
- Societies closer to the equator.
- Low reliance on fisheries.
- Low reliance on renewable energy.
- Large forested areas.
- Industrial base able to produce advanced resilient foods.
- Societies with coast lines in the Pacific for seaweed farming.

For nuclear winter, additional factors are:

- Not being a nuclear weapon state, not being in a military alliance with a nuclear weapon state and not being in conflict with a nuclear weapon state.

For volcanoes, additional factors are:

- Distance from active volcanoes or volcanoes that had major eruptions in the past.
- Strong volcanic monitoring capacity and institutional preparedness.
- Low dependence on regions considered a “pinch point” where critical infrastructure or trade routes intersect with volcanic regions (highest risk: South‐East Asia, the Mediterranean, and western North America).
- Low dependence on monsoon‐driven agriculture, as large eruptions can disrupt major monsoon systems and ENSO.

For near‐Earth objects, additional factors are:

- Low proportion of population near the low‐lying coastal areas, to avoid large tsunamis after impact.

When we combine these factors with the general factors from Section 3.1.6, it becomes even more difficult to find any society that could fit this description, as the factors have inherent tensions:

- Islands maximize isolation but often lack industrial capacity and rely on trade.
- Many islands have volcanoes.
- Islands have long coastlines and are often low‐lying making them more exposed to tsunamis and less asteroid resilient.
- The Southern Hemisphere is less vulnerable, but the most industrialized nations are Northern.

Still, there are some societies that fit these factors better than others. Across all three ASRS categories, Australia seems to be the best. It is located in the Southern Hemisphere, far away from nuclear conflicts (though it is allied with the US, which might increase risks), it did not experience volcanic eruptions in the Holocene, but a large interior, which people could relocate to if an asteroid strikes (although a large fraction of this is desert). Additionally, it is a major food producer, has diverse climate zones, a long growing season, significant coal and gas infrastructure, large forested areas and a Pacific coastline. Tasmania might even be considered a refuge in a refuge, as it is part of Australia, but also an island, with large agricultural production and a temperate climate.

The next best fits could be Argentina, Brazil and New Zealand, especially if they collaborate with Northern Hemisphere countries for crop relocation [128]. They are all (mostly) in the Southern Hemisphere, fairly remote, and produce a large amount of food. However, New Zealand has active volcanoes, a small industrial base and is entirely surrounded by water and thus vulnerable to tsunamis. Brazil and Argentina score better on all of these metrics, but have high levels of inequality and historical political instability. Other countries with a favorable set of resilience characteristics include Uruguay (very democratic, Southern Hemisphere, produces food, but low population and thus small (but well developed) industrial base) and Chile (long Pacific coastline for seaweed farming, Southern Hemisphere, some climate diversity, but active volcanoes, high reliance on fisheries and high trade dependence). One country with very conflicting results is Iceland, as it checks many boxes (like being an island or having experience with volcanoes), but it also lies relatively far north, which means it would suffer a significant temperature shock in ASRS.

### 3.3 Global Catastrophic Infrastructure Loss

**FIGURE 5:**
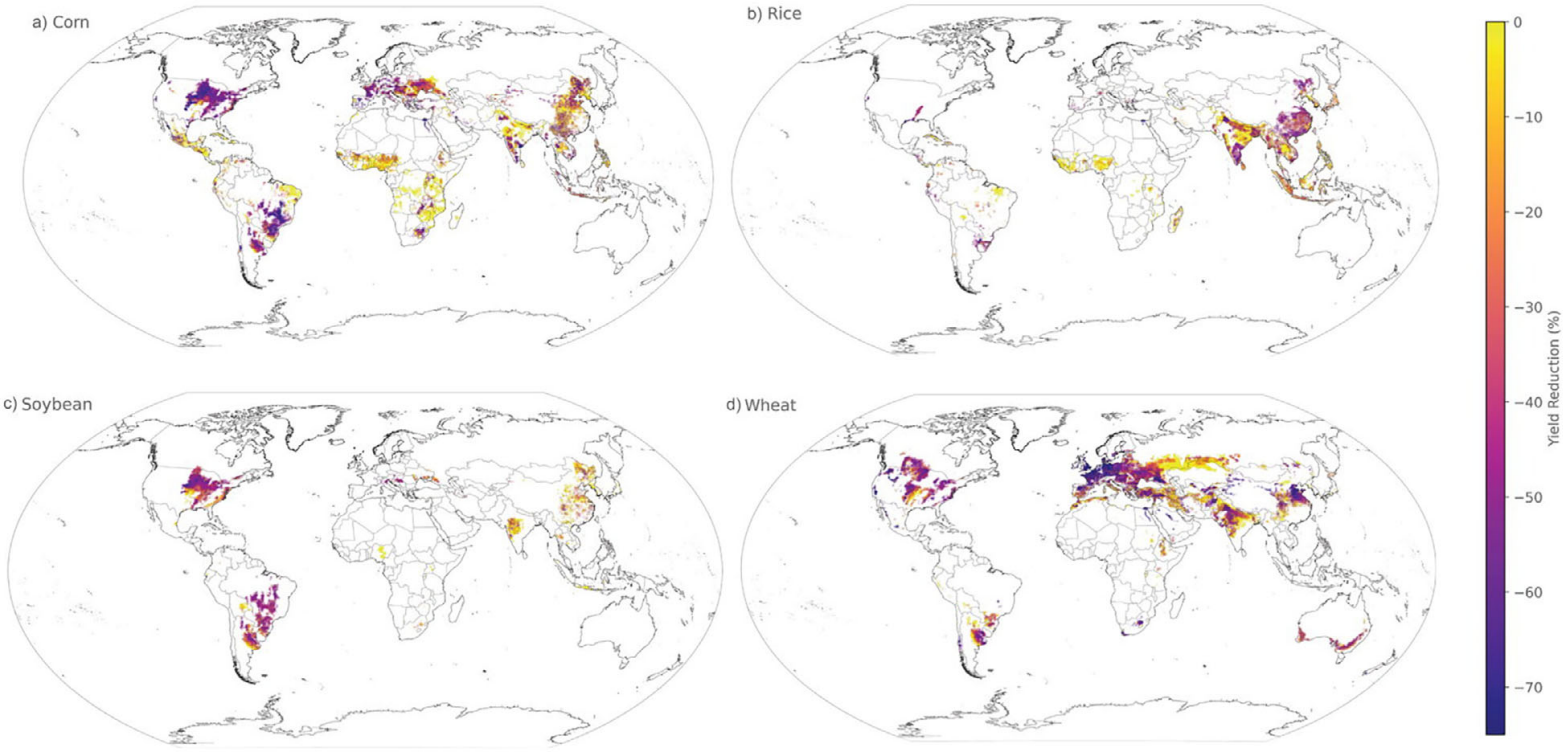
Global yield decline for corn, rice, soybean and wheat after GCIL. Based on Moersdorf et al. [20], as adapted by García Martínez et al. [19].

In the papers we identified, the most straightforward way to increase resilience to GCIL is low reliance on more elaborately manufactured technology and complex supply chains. The more a society uses digital technologies, especially in their infrastructure, the more vulnerable it becomes to any catastrophe that disrupts these technologies [129]. This could be especially true for agriculture. Regions which currently use few agricultural inputs like fertilizers or pesticides are expected to maintain their current food production levels, even after infrastructure collapse [20]. This is fulfilled by many smallholder farmers globally, but also by alternative kinds of agriculture in more industrialized societies (e.g., organic farming or permaculture) (Figure 5).

Local resource availability also builds resilience. This is especially true of large amounts of wood, which can be used as energy directly or after gasification [130]. These local resources could be stretched further if a stockpile of critical industrial goods exists which can be used as a buffer until local substitutes are found [131] and by gaining access to unusual local resources like nutrient extraction from leaves [70, 132].

A significant risk in GCIL is disruption to the electrical grid. A more robust grid can be accomplished in several ways. If enough warning time exists before the damaging event, the grid can be turned off for the duration and restarted afterward, as a deactivated grid is much less likely to be damaged [133]. Physical protection like a more resilient grid topology can be introduced [134]; an example of this is the highly interconnected European grid with many cross border links, so countries can help each other out in case a disruption happens [135]. However, it can also be a vulnerability, as shown with the 2025 blackout in Spain and Portugal. The grid can be stabilized by having more physical inertia in the form of moving infrastructure (like moving turbines), which is less present in grids more focused on renewables [136] and a more developed black start capability [137].

In parallel to a disruption of the electrical grid, a large risk is also the disruption of longer distance communication. This is essential to ensure coordinated response and even the restart of the electrical grid is reliant on communication [33]. Therefore, it would be prudent to have backup communications. This could include EMP‐hardened satellites or large networks of shortwave radios [138]. This likely exists at least for some militaries, but less so for the civilian sector.

Also, disruptions of production do not scale linearly with industrial output decline. For example, during World War II Japan's industrial capacity was destroyed to around 30%, which led to a drop in production of around 80% [131].

Finally, decentralization provides resilience, as local hubs are more capable and there is not a single point of failure. This can be decentralization of storage of foods and fuel [33], decentralization of the electrical grid, especially when it is more focused on renewable energy and the ability to form microgrids [33, 34].

In addition to this “traditional” infrastructure which can be disrupted, in the modern world, there is also globally critical infrastructure. These are parts of the infrastructure, which a country relies on, but which are located in other countries or in other regions like space or the oceans. Examples of this are GPS, the Panama Canal or the Svalbard Global Seed Vault. If this kind of infrastructure is disrupted it can have massive consequences as well, but are even harder to recover from, as they are not easily accessible if they are beyond one's borders [139].

#### 3.3.1 Geomagnetic Storms

Geomagnetic storms can be predicted and grid protection measures introduced ahead of time. Only a few countries possess this capability, including the US, EU, UK, Japan, Australia, France, and China. Partly this information is shared, but it is best to be self‐sufficient in this information, as it gives quicker and easier access [133]. Fry [140] argues that the United States and European Union have been spending a relatively high amount to become more resilient against geomagnetic storms by investing in better forecasting. Additionally, the grid can be hardened against geomagnetic storms, which has for example been implemented by Canada, the US and Sweden [134]. Though public information on how much of the grid this affects and to what extent is scarce.

**FIGURE 6:**
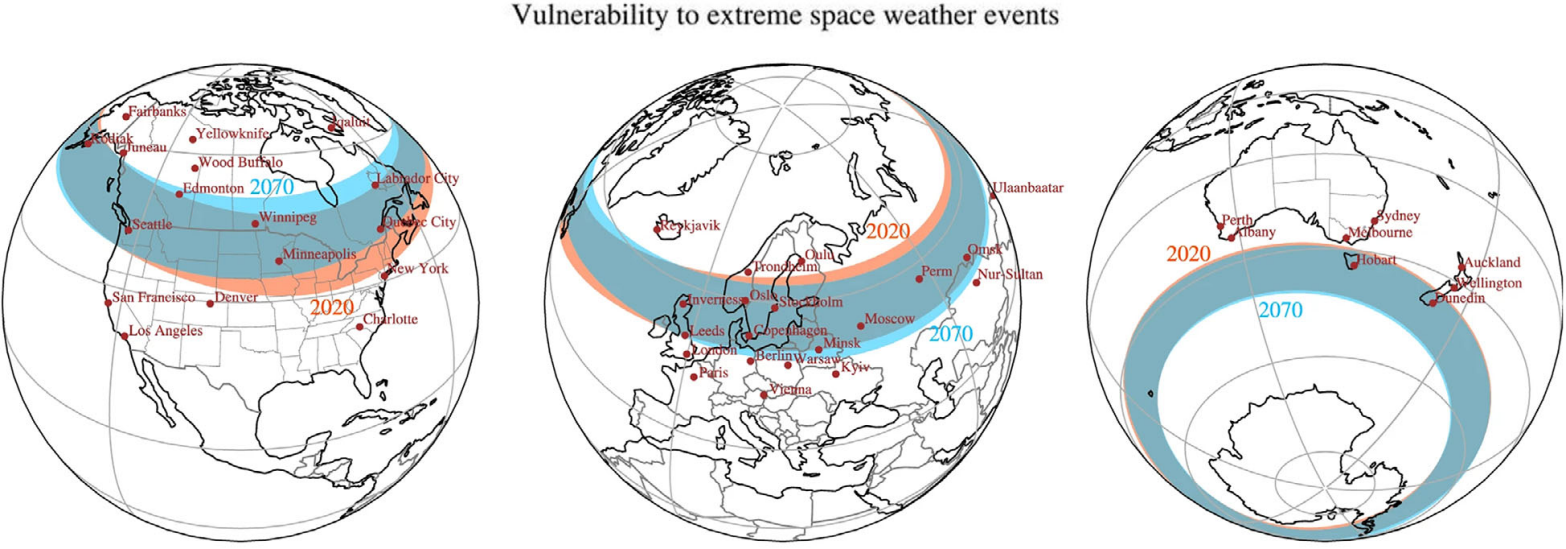
Zones for 2020 and 2070 with the highest likelihood of damages from space weather. Originally from Maffei et al. [141].

Geomagnetic storms do not hit all places the same, there are preferential zones. This means damage outside these zones is less likely [141]. The most exposed are Canada, Ireland, United States, United Kingdom, Northern Germany, Baltics, New Zealand, Tasmania, Russia and Scandinavia (Figure 6). However, larger storms might reach further, potentially hitting most places on Earth, albeit not all at the same time, as due to Earth's rotation different places are more or less exposed over time. Further, independently from these satellites will be disrupted by large storms as well, meaning that countries with a high reliance on satellites will incur more damages.

How dangerous these storms are is still actively debated, with estimates ranging from a big, but manageable disruption [133], to estimates that this could destroy around 10%–15% of the global economy (Schulte in den [142]).

#### 3.3.2 High Altitude Electromagnetic Pulse

HEMP can be caused by detonating a nuclear warhead at a high elevation. Thus states not in conflict with hostile, nuclear‐armed states are unlikely to be targeted. In general, HEMP effects cover a wide area, typically as far as the horizon, with effects diminishing based on distance [143]. So, states proximate to potential HEMP targets might be affected, especially when the potential target state is small, because the effects are more likely to extend beyond national borders. Critical infrastructure and military systems are vulnerable to HEMP effects and could be massively disrupted [144]. Because the effects of HEMP can be so broad and varied, increasing resilience can come at high costs, and many systems may remain unprotected [35, 144].

Several states have undertaken efforts to protect military and civilian systems from the EMPs, including the US, UK, Russia, China, South Korea, and Taiwan [145]. These are typically states that face plausible nuclear threats. However, states without dedicated HEMP resilience programs can develop some resilience through alternative means. Activities to increase resilience against lightning strikes and geomagnetic disturbances increase resilience to HEMPs, due to some common waveforms. The waveforms generated from an HEMP have three time‐based components: E1, E2 and E3 [146]. The E2 waveform bears strong similarities to lightning strikes, while the E3 waveform bears strong similarities to geomagnetic disturbances (Department of Energy, 2023), meaning states with protective measures against those hazards will have some resilience.

Occasionally, states have explicitly chosen to focus less on resilience in favor of prevention, such as the UK [147]. Preventative activities include international treaties, norms, export controls, counter‐proliferation activities and disarmament efforts (that is measures to reduce or even eliminate nuclear weapons) [148].

#### 3.3.3 Cyberattacks

Societies with an older electrical grid are likely to be more resilient to large cyber attacks, as they do not have the digital systems which could be hit by a cyber attack. This has been showcased by Ukraine, which was able to revert its electrical grid to manual control after its digital components were disrupted by Russian attacks [149]. During large cyber attacks it could be highly helpful if the system can be switched into manual control, instead of relying on digital technology controlling the system. Generally, the more modern the grid is which a society has, the less likely it is to have this ability to switch to manual control. This could be partly mitigated by hardwiring the control systems, as this means they cannot be changed remotely [149]. Alternatively, a well developed cyber defense could prevent large‐scale cyber attacks before they can create damage [150].

#### 3.3.4 Summary of Resilience Factors Relevant for Global Catastrophic Infrastructure Loss (GCIL)

In addition to the general features identified in Section 3.1.6, GCIL resilience appears to be increased by the following factors:

- Low reliance on digital technology.
- Agriculture less reliant on artificial fertilizers and pesticides, like smallholder farmers or organic agriculture.
- Abundant local resources, especially wood and stockpiles of critical industrial goods.
- A hardened electrical grid, with distributed generation, many interconnections, the ability to ‘island’ parts of the grid, a good black start capability, and high physical inertia.
- Decentralized organization in as many areas of society as possible.

For geomagnetic storms, additional factors are:

- Good forecasting capability of space weather (available in the United States, European Union, United Kingdom, Japan, Australia, France and China).
- Location less likely to be impacted by severe space weather (this excludes Canada, Ireland, United States, United Kingdom, Northern Germany, Baltics, New Zealand, Tasmania, Russia and Scandinavia).

For HEMP, additional factors are:

- Being a non‐nuclear weapon state which is not in NATO and not in any military alliance with nuclear states.
- Having highly hardened infrastructure (which nobody has on a large scale, there are only highly localized examples).

For cyber attacks, additional factors are:

- An electrical grid which can be run in manual mode, more likely for older electrical grids.
- Well‐resourced and effective cyber security.

When we combine these factors with the general factors from section 3.1.6, we see few locations exhibit good resilience, as the factors create inherent tension. Society's resilience is described as being increased by being:

- High tech and highly industrialized to be better able to prevent collapse, but also low tech, to more easily adapt to post catastrophe conditions.
- Decentralized on all levels, while also having effective central control of a large industrial base, or strategic responses to catastrophe.
- Highly interconnected but also very isolated.

Societies that aim to be more resilient always have to make some compromise, but we can still identify some societies that likely have a higher resilience than others. The best picks here are again Australia and New Zealand, if they are able to maintain trade and cooperation. They are both remote and democratic and have relatively low inequality. Australia has a good industrial base and both are food producers. New Zealand even produces its own nitrogen fertilizer and phosphorus stocks in soils could be enough for years to decades without much extra input. Additionally, Australia has good geomagnetic storm forecast capabilities, but could be higher up on a nuclear target list, due to its alliance with the United States. Other national‐level jurisdictions which could offer a good compromise are Switzerland and Uruguay. Both are highly democratic, have low inequality and a grid with high inertia, but not import dependent, due to hydro power. Switzerland might be collateral damage in case of a HEMP in Europe, but it also has a partly hardened infrastructure. Uruguay has a limited, albeit well‐rounded industrial base, due to its small size, but it is far away from potential HEMP targets and geomagnetic storms danger zones. If we exclude stable democratic structures, China and Brazil fit many of the factors as well. Also, Cuba is a one party authoritarian state, but exhibited resilience in contexts of limited trade and reduced availability of agricultural inputs such as fertilizers, pesticides and liquid fuel. France and Japan are also potentially resilient, at least to geomagnetic storms, as they have good space weather forecasting abilities.

### 3.4 Global Catastrophic Biological Risk

GCBR resilience has the largest amount of literature, likely due to Covid‐19, as most studies we obtained examined resilience in this pandemic. A handful (e.g. [21, 151, 152, 153, 154]) addressed pandemics or biological risks more generally. Due to the larger number of sources, we have split this section into: (1) health security and health system factors, (2) demographic and geographical factors, and (3) governance, societal, and response factors.

#### 3.4.1 Health Security and Health System Factors

Many studies link components of general health security to better pandemic outcomes, implying resilience to GCBRs. These include: health system financing [155, 156, 157], resulting in effective health facilities and capacity, in terms of hospital beds, health workforce, health access and quality [158, 159, 160]. Also, strong public health infrastructure [151], including testing and health surveillance facilities and capabilities [161], health data management, data sharing, and data infrastructure [156, 162, 163]. Also, vaccine programmes and availability [151], and preexisting biosecurity capability [164]. Universal healthcare is also cited as a protective factor [165].

**FIGURE 7:**
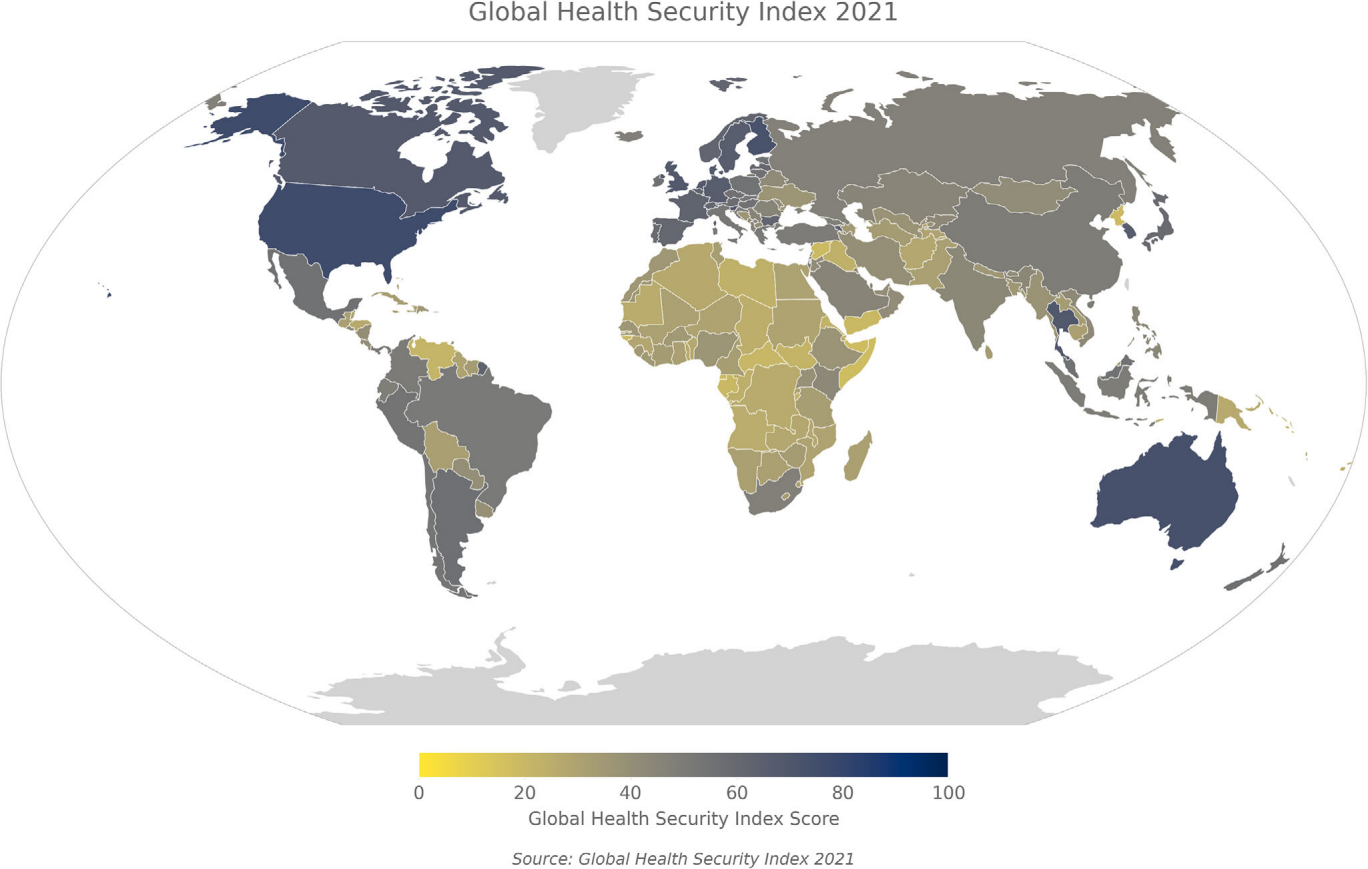
2021 GHS Index scores reflect actions and investments taken by jurisdictions in response to Covid‐19 and therefore some of the resilience factors for future pandemics (ghsindex.org). Higher means better.

Our literature search was not a systematic review of health system capabilities specifically, but the health system factors just mentioned, along with others, have been measured systematically in various ways. The most cited metric of overall health security in the documents identified is the Global Health Security Index [166]. First established in 2019, prior to the Covid‐19 pandemic, this is a comprehensive, factors‐based assessment of health security capabilities across 195 States Parties to the International Health Regulations. The metric encompasses six categories and quantifies societies’ abilities or potential to carry out public health functions necessary for infectious disease outbreak prevention, detection and response, by giving an ‘Overall Score’ out of 100. This approach has limitations like being too light on social political aspects like governance or leadership [14, 158, 167], but generally seems to have been a good predictor of excess mortality [14, 168, 169], especially in non‐islands [14]. This highlights especially the African societies as highly vulnerable to pandemics, but also the middle east (Figure 7).

#### 3.4.2 Demographic and Geographic Factors

Demographic factors identified as increasing risk in the literature we sourced included: population age structure [158, 170, 171], though the exact nature of this varies by pandemic [151], higher rates of obesity [171], higher cardiovascular disease and smoking rates [170]. Population density (and especially dense living situations) appears to affect the number of cases [160], but not necessarily the death rate [159].

Wealth and conflict are also important factors for resilience to the impact of pandemics. Developing societies [151] and societies suffering conflicts [162], are more impacted when pandemics strike. Societies dependent on imports of critical supplies such as food and energy are likely also vulnerable [172], while subsistence farmers and small remote communities may exhibit resilience [153].

Island nations have long been considered to have some protection against biological threats [173], especially when they are able to strictly control their borders. This was borne out during the Covid‐19 pandemic when islands suffered far lower excess mortality than non‐islands (64.8 vs 194.3 deaths per 100 000) [14, 160, 174]. This is despite their generally poorer health security, for example in terms of GHS Index scores [168].

Other geographical features associated with likelihood of better pandemic outcomes include: reduced exposure to wildlife habitats [163], remote countries like Bhutan or Iceland [152, 156, 175], locations away from transportation networks [151, 163], with little air travel connectivity [156], few foreign visitors or international connections [170], islands with fewer travel connections [176] and limited entry points [177]. Previous exposure to pandemics and biological threats appears protective [174]. The experiences of China and Western Australia show that some well‐organized non‐island jurisdictions can also effectively limit pandemic harms over long periods [164].

#### 3.4.3 Political and Societal Factors

Political and societal factors associated with better preparedness and more successful pandemic responses include higher GDP per capita and lower rates of poverty [151, 153], strong, transparent, effective governance [157, 168, 171, 178], with strong democracy [62]; Ramírez [179], including data‐driven decision‐making [160]. Broader economic and social well‐being also matters: low income inequality is associated with better health outcomes, alongside adequate social safety nets and high personal income. This relationship between lower inequality and better health holds even after accounting for absolute poverty and per capita income [180, 181].

Additional factors include high‐level political commitment [162], decisive leadership [158], and trust, public confidence, and respect for social rules and institutions [160, 162, 171], as well as good social cohesion [174]. In part, the poor Covid‐19 outcomes in the United States [182] and Latin America (Ramírez [179]) appear due in part to a lack of political will and poor social cohesion. This picture gets complicated by societies tending to become more authoritarian during and after crises [183], which might generally decrease their resilience.

Less corruption, greater government effectiveness, higher regulatory quality and stronger rule of law were associated with fewer deaths and greater vaccine coverage during the Covid‐19 pandemic [174], as were participatory, rather than authoritarian regimes [165]. However, communication effectiveness could be important [160], as is the ability to maintain economic activity while limiting human movement [164]. Jurisdictions implementing an ‘exclusion/elimination’ strategy, often in combination with geographic advantages such as island status, exhibited much lower excess mortality during 2020–2021 (−2.1 vs 166.5 deaths per 100 000) when compared to jurisdictions not implementing this strategy [184]. Additionally, border control factors such as duration of border closure were associated with fewer Covid‐19 deaths in islands but not in non‐islands [184]. Delaying the onset of effects is also associated with reduced overall impacts [169].

Beyond healthcare system quality and preparedness [159], other infrastructure identified that could aid resilience to pandemics includes: supply chain capacity in terms of medicines and technologies [154, 163, 178], logistics and transportation networks [172], communication infrastructure [172, 178], decentralized infrastructure and distributed systems, heterogeneous food supply systems [153], and effective and resilient food supply and distribution systems [172].

All these factors likely help explain the imperfect alignment between GHS Index scores (or other metrics of health security) and observed pandemic outcomes, which depend additionally on geographic, demographic, political and societal factors.

#### 3.4.4 Summary of GCBR Resilience Factors

In addition to the general features identified in Section 3.1.6, GCBR resilience could be increased by the following factors:

- Better health system in general in terms of financing, capacity and infrastructure.
- Universal healthcare.
- A higher value of the Global Health Security Index (the highest values are in Canada, the United States, Spain, France, United Kingdom, Germany, Denmark, Finland, Norway, Sweden, Thailand, Australia, New Zealand, Japan and South Korea).
- A more healthy population.
- Being an island or very remote, especially if tight border control is enforceable.
- Previous experience with biological threats.
- Public confidence and respect for social rules and institutions.
- Less corruption and solid rule of law.
- Secured supply chains.
- Decentralized systems (in most cases).

These factors conflict less with the general factors from Section 3.1.6 than for the other GCRs but there is still some tension:

- Universal health care and strong health systems require substantial resources, which are usually not available in countries which are remote.

There are several societies which check most of these boxes. Australia and New Zealand are both democratic, low corruption, provide good health care, have low inequality and can easily enforce closed borders. However, they are both quite trade dependent and New Zealand only has a small industrial base. Besides these two countries, Scandinavia, Canada and Switzerland also satisfy almost all factors, apart from being remote. Switzerland might be better able to isolate itself due to the mountainous terrain, but it is quite small and more dependent on trade than the others. Norway could be especially good, as it is relatively energy independent due to its oil reserves and large hydropower capacity. Japan and South Korea also tick many boxes, but are very trade dependent and have aged populations which could be more susceptible to certain diseases, such as Covid‐19. Iceland also has potential here, but also has a very small economy and is very trade dependent.

## 4 A Cross GCR Comparison

**FIGURE 8:**
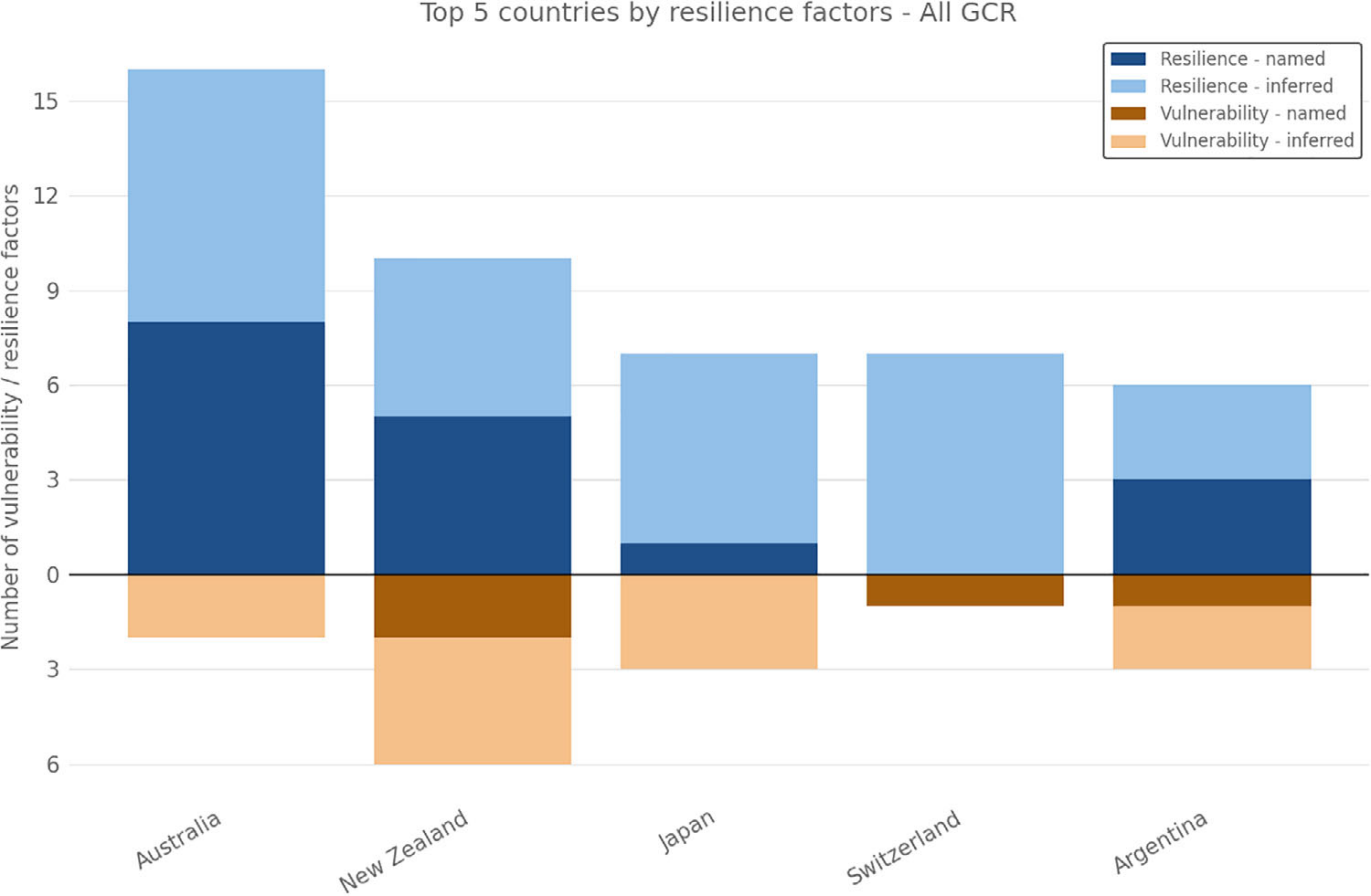
Overview of how often countries were mentioned directly as resilient or vulnerable in cited documents or inferred by the authors of this paper based on factors described in other documents.

Bringing all these insights from the literature together, we can create a summary of which countries are either named directly in the documents considered or inferred from resilience factors mentioned in this paper (Figure 8). This is not a systematic, empirical selection, but meant to highlight countries that came up often in the documents discussed above (e.g. Australia).

Australia is likely the most resilient place on Earth when it comes to GCR. It is the country most often mentioned in the documents and has many factors that could enhance its resilience. New Zealand and to a lesser extent Switzerland, Japan and Argentina are also mentioned often, but also have many vulnerabilities highlighted. Australia, New Zealand and Japan are the only countries which have resilience evidence (named + inferred) across all three GCR categories, while only Australia is directly named across all three.

Yet even those countries are weak against certain hazards. Many of the factors describing New Zealand's vulnerability are also shared by many other countries, they are only mentioned directly for New Zealand, as it is also one of the most studied countries in relation to GCR. All of the most mentioned countries except Switzerland have long coastlines which could be impacted by tsunamis from a near‐Earth object. All except Switzerland could be impacted by one of the several volcanoes in South‐East Asia and South America erupting, as Tambora did in 1815. All are reliant on trade. There is no place on Earth which is a complete refuge from GCR.

**FIGURE 9:**
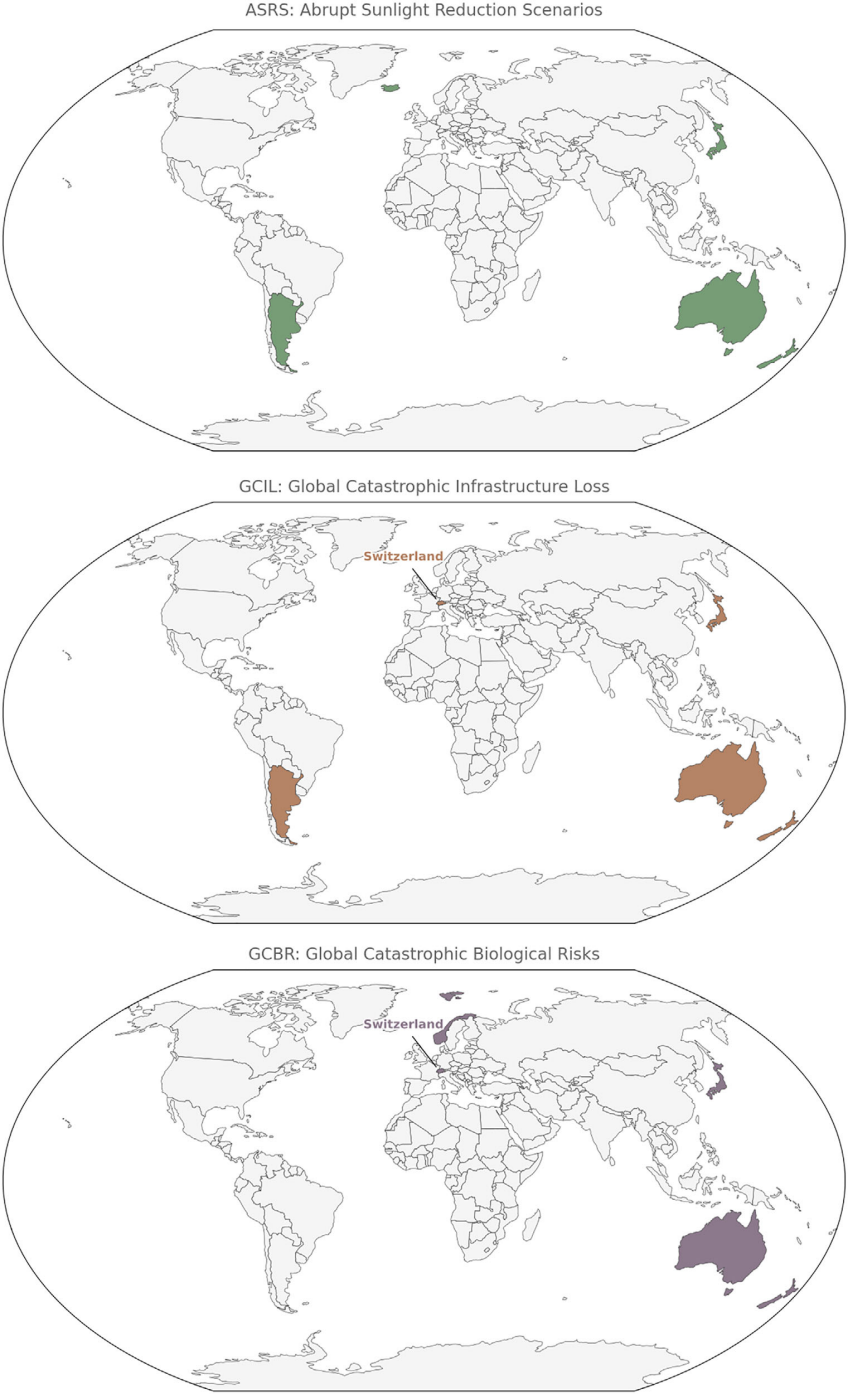
Visualization of the five countries which are mentioned most often as resilient in cited documents or inferred by the authors of this paper based on factors described in other documents for the three kinds of GCR considered. Switzerland is highlighted for better visibility.

But still we can see that for each of the GCRs considered here there are several countries that could fare better than others. Examples of such countries can be found on almost all continents (Figure 9). These tend to be middle to high income countries. Most of them are democratic and have low wealth inequality. ASRS favors the Southern Hemisphere, GCIL countries with the wealth to invest in resilience measures like geomagnetic storm prediction, GCBR might best be endured by islands or in countries with a strong healthcare system, like in Norway or Japan.

There are no countries except Australia, Japan and New Zealand which show up in the top 5 most mentioned countries across all three major GCR categories (Figure 9). This highlights trade‐offs inherent in GCR resilience. Many of the factors that make a society more resilient against one kind of GCR, make it less resilient against another. For example, being able to isolate a society to protect it from pandemics, also implies that it likely could be more on its own in case of a GCIL. This is similar to the problem highlighted in the previous section that many of the resilience factors in the literature are contradictory, making it ambiguous to highlight specific places as most resilient.

Besides the countries discussed here directly, there are also other countries which have some GCR resilience, but just have not been studied much from this angle. An example here could be Uruguay which also tracks well for many of the resilience factors discussed.

## 5 Discussion

### 5.1 Trade‐Offs in Resilience Factors

There is no single place on Earth which is simultaneously resilient against all kinds of GCR. This reflects the trade‐offs between resilience factors, see Table 1 for all trade‐offs identified in this study. This trade‐off happens on several levels, but arguably the most important are:

- *Across GCR categories*: Many factors that make a society more resilient against one kind of GCR, make it more vulnerable against another. For instance, geographic isolation makes it easier to isolate a society during a pandemic, but it also means it is far away from help and partners in case of GCIL. Similarly, a large industrial base offers more opportunities to start producing resilient foods during ASRS, but also means that the society is likely more reliant on digital infrastructure and thus more vulnerable to GCIL.
- *Between mitigation and recovery*: Factors that can prevent a crisis from occurring, can mean that if a crisis does occur it will be worse and recovery will be more difficult. The opposite is also true: factors which lead to shorter‐term vulnerability may buffer a society from a catastrophe and allow for a swifter recovery. For instance, smallholder farms are likely less affected by GCIL, but their lower yields mean that less food is available in the first place and thus the system could be more vulnerable to disruptions. Also, they might recover more quickly after the disruptions happen, as their livelihood is not disrupted as much as in more urbanized regions.

**TABLE 1:** Main trade‐offs and tensions identified. ASRS = Abrupt Sunlight Reduction Scenarios; GCIL = Global Catastrophic Infrastructure Loss; GCBR = Global Catastrophic Biological Risk; NEO = Near‐Earth Object; HEMP = High Altitude Electromagnetic Pulse.

Factor A	Factor B	Nature of the trade‐off	GCR categories affected
***Geographic and locational***			
Geographic isolation	Access to trade and aid	Islands can seal borders during pandemics but are far from help during GCIL, often lack industrial capacity, and typically cannot sustain resource‐intensive health systems that favor GCBR resilience.	GCBR vs. GCIL
Southern Hemisphere location	Industrial capacity	Less ASRS cooling and no nuclear‐armed states, but most industrial capacity is in the Northern Hemisphere.	ASRS vs. GCIL
Island status	Volcanic exposure	Many islands offering isolation (e.g., New Zealand, Indonesia) also have active volcanoes.	GCBR vs. volcanic ASRS
Island status	Tsunami exposure	Long coastlines useful for isolation also increase exposure to tsunamis from asteroid impacts.	GCBR vs. NEO impact
Nuclear alliance membership	Targeting risk	Nuclear umbrellas provide deterrence but elevate the risk of being targeted in nuclear war or HEMP.	ASRS, GCIL vs. Security
***Infrastructure and technology***			
Large industrial base	Digital dependency	Enables resilient food production during ASRS but implies greater reliance on complex supply chains vulnerable to GCIL.	ASRS vs. GCIL
Modern, interconnected systems	Low‐tech adaptability	Digital and interconnected systems aid surveillance and mutual grid support, but increase vulnerability to cyber attacks, HEMP, and cascading failures (e.g., 2025 Spain–Portugal blackout). Older grids can revert to manual control.	GCBR vs. GCIL
Renewable energy	ASRS energy supply	Wind and solar output decrease during ASRS. Renewable‐heavy grids also have less physical inertia, reducing stability during GCIL.	ASRS, GCIL vs. sustainability
***Food system***			
Conventional agriculture (high yields)	Low‐input agriculture (input robustness)	High yields provide a larger food buffer, but depend on fertilizers and pesticides that become unavailable during GCIL.	ASRS, GCIL
Sustainability‐oriented food systems	Food system buffer and resilient food capacity	Sustainable farming and reduced meat production benefit planetary boundaries, but lower yields and loss of the latent food reserve in livestock could worsen outcomes during ASRS.	ASRS vs. sustainability
***Governance and organization***			
Centralized coordination	Decentralized resilience	Central control aids strategic crisis response; decentralization avoids single points of failure and fosters innovation.	All GCR
***Demographic and structural***			
Urban concentration	Supply chain vulnerability	Cities concentrate industrial and health capacity but depend on continuous imports and collapse quickly when supply chains fail.	All GCR

These trade‐offs also make it difficult to simply rank countries by a composite measure of their GCR resilience since this would involve weighting these trade‐offs against each other. Is low inequality more important than domestic fertilizer production? Is island status more valuable than high state capacity? We cannot answer these questions with the available evidence. Many are also values‐based issues which cannot be answered by a technical analysis. What we can do is distinguish between factors that are fixed and those that are amenable to policy intervention (Table 2), and identify the factors that incur the fewest cross‐GCR trade‐offs. To make sure that such trade‐offs are done in a fair and just manner, deliberative democratic processes like citizens’ assemblies could be used.

**TABLE 2:** Fixed versus modifiable resilience factors. ASRS = Abrupt Sunlight Reduction Scenarios; GCIL = Global Catastrophic Infrastructure Loss; GCBR = Global Catastrophic Biological Risk; NEO = Near‐Earth Object; HEMP = High Altitude Electromagnetic Pulse.

Category	Factor	Fixed or modifiable?	Relevant GCR	Example policies
Geography	Island status/remoteness	Fixed	All (esp. GCBR)	—
	Southern Hemisphere location	Fixed	ASRS	—
	Distance from active volcanoes	Fixed	Volcanic ASRS	—
	Proportion of low‐lying coast	Fixed	Tsunami caused by near‐Earth object impact	Managed retreat, elevated critical infrastructure
	Climate zone diversity	Mostly fixed	ASRS	Landrace seed banking, crop diversity programmes
	Forest cover	Partly modifiable	ASRS, GCIL	Reforestation, agroforestry
Governance	Democracy/political participation	Modifiable	All	Democratic institution strengthening, anti‐backsliding measures
	Inequality	Modifiable	All	Progressive taxation, social safety nets, land reform, wealth taxes
	State capacity	Modifiable	All	Civil service reform, emergency management investment
	Corruption/rule of law	Modifiable	All (esp. GCBR)	Anti‐corruption institutions, judicial independence
	GCR‐specific policy structures	Modifiable	All	Parliamentary commissioners for extreme risk, GCR in national risk registers
	Social cohesion/trust	Partly modifiable	All (esp. GCBR)	Inequality reduction, participatory governance, civic education
Production & infrastructure	Industrial base size	Modifiable (slowly)	All (esp. ASRS)	Industrial policy, strategic manufacturing retention
	Agricultural diversity & self‐sufficiency	Modifiable	ASRS, GCIL	Crop diversification incentives, strategic reserves
	Food stockpiles	Modifiable	All	National strategic food reserves, pre‐positioned stockpiles
	Grid hardening/decentralization	Modifiable	GCIL	Microgrid investment, black start capability, grid topology upgrades
	Backup communications infrastructure	Modifiable	GCIL	EMP‐hardened satellites or large networks of shortwave radio
	Resilient food production capacity	Modifiable	ASRS, GCIL	R&D in alternative proteins, seaweed farming, leaf protein extraction
	Cyber defense capability	Modifiable	GCIL (cyber)	Manual override systems, hardwired controls, cyber defense
	Health system capacity	Modifiable	GCBR	Universal healthcare, health workforce investment, surveillance infrastructure
Preparedness	Emergency planning for GCR	Modifiable	All	National GCR emergency plans, international cooperation agreements
	Space weather forecasting	Modifiable	GCIL (geomagnetic)	Investment in monitoring capability
	Seed banking	Modifiable	ASRS	Expand seed vault participation, maintain landrace diversity
	Border control capacity	Modifiable	GCBR	Entry point reduction planning, quarantine infrastructure
	Fuel and medicine stockpiles	Modifiable	All	Strategic reserves, domestic production capability for essentials

### 5.2 Which Factors Offer the Most Policy Leverage?

Not all relevant factors can be changed (Table 2). A country cannot suddenly become an island or relocate to the Southern Hemisphere. But many of the factors identified are concerned with the structure of a given society and how well it has prepared itself. These can all be changed, given enough political will. Some of those have few trade‐offs between different GCRs and should thus be prioritized:

- Governance quality: Democratic institutions, low inequality, state capacity, low corruption, and social cohesion appeared as resilience factors for every GCR category examined. They also do not trade‐off against each other, but instead reinforce each other. It is encouraging that factors that have been hypothesized to increase resilience like state capacity [51, 52], higher social cohesion [48, 49, 50], more transparent government [52, 185] and lower inequality [68, 186, 187, 188], all turned out to be important factors when it came to the response to Covid‐19 [14, 62]. Also, these factors carry other widely documented societal benefits independent of any catastrophe.
- Decentralization: This factor also appeared across categories with few trade‐offs, though it can conflict with the need for centralized coordination during acute crises. However, this could be partly circumvented by delegating more power to lower levels of government. Decentralization could be achievable through policy, microgrids, distributed food reserves, and diversified agriculture, all of which do not require geographic relocation.
- GCR‐specific preparedness: Currently, most countries invest little to no resources to prepare for GCRs. Many measures could be implemented here. This includes incorporating GCR into national risk registers [15], developing national emergency plans that account for global‐scale disruptions [20], investing in resilient food research and production capacity [19, 55], and establishing pre‐catastrophe international cooperation agreements that address trade maintenance after catastrophe [13, 19]. Such actions could likely also help in catastrophes on a scale smaller than a GCR and could have benefits for other national goals like improved healthcare or reduced emissions.
- Food system resilience: While there is some tension between maximizing yields (conventional agriculture) and maximizing robustness to input loss (organic/low‐input systems), there are policies that reduce vulnerability across scenarios without prohibiting trade‐offs, such as maintaining a diverse portfolio of agricultural approaches and investing in resilient food technologies (single‐cell protein, seaweed, leaf protein extraction).

Concerningly, several of the most important modifiable factors are currently trending in the wrong direction. Democracies are in decline globally [189, 190]. Inequality is rising for the majority of the world's population [69]. Supply chains are increasingly concentrated and globalized [89]. These trends simultaneously reduce resilience to multiple GCR categories.

### 5.3 Comparing GCR Resilience to Other Conceptions of Resilience

The resilience factors identified here align with established principles in resilience science. Widely used principles here are diversity, redundancy, modularity, and connectivity [12, 191]. They all appear in our findings, though sometimes under different labels. Diversity maps onto our findings about agricultural diversification, diverse climate zones, and diversified trading partners. Redundancy appears in the importance of stockpiles, large industrial bases with repurposing potential, and multiple food production pathways. Modularity corresponds to our findings that decentralization could often make societies more resilient to GCR. Connectivity is present but ambivalent: trade connectivity might be both a resilience factor (access to diverse resources) and a vulnerability (cascading failure), consistent with the broader resilience literature's recognition that connectivity has thresholds beyond which it transmits rather than absorbs shocks [192]. Where our findings diverge from standard resilience thinking is in the emphasis on self‐sufficiency and isolation. Conventional resilience frameworks tend to emphasize connectivity and openness as positive attributes. In the GCR context, the ability to disconnect can be protective.

This paper is not the final answer on what societies are most resilient to GCR, not only because this is in constant flux, but also because it is an initial, broad overview, one which future research can build on. For example, this could lead to a global catastrophic risk resilience index, which would create a more quantified assessment and cover more factors than a first attempt at that by the Global Governance Forum [86]. As mentioned earlier, it is difficult to directly compare many of the factors here. However, it might still be worthwhile to create sub‐indices, separated by hazard and also potentially by prevention, immediate response and recovery. This more finely grained approach would more clearly highlight which countries and regions are most resilient and could be further developed by creating a composite score with weights that can be changed, to reflect differences in resilience.

Similarly, a more in depth comparison with existing measures of resilience could showcase how general resilience factors and GCR resilience factors overlap or diverge. For example, World Risk Poll 2024 also identified Australia and New Zealand as a very resilient place, but some of the countries that have many factors of GCR resilience like Argentina or Uruguay only get a low score in their assessment (Lloyd's Register [193]).

### 5.4 Structural and Economic Dimensions

Many of the problems identified in this study are also linked to structural features of how economies are organized. Market‐based systems without strong public coordination mechanisms tend to systematically underprice diffuse, high‐uncertainty, long‐horizon, risks. This is because the costs of inaction are deferred while the costs of mitigation are immediate. This is most clearly visible for climate change [194], but also relevant for the GCRs discussed here. Such effects are compounded by the concentration dynamics that capitalist markets tend to produce, including the dominance of few crops, few major corporations, and few exporting nations, which reduces the redundancy and diversity that resilience requires [89]. More specifically, the neoliberal policy turn of recent decades, characterized by deregulation, privatization, and the erosion of state capacity, has weakened the public institutions that resilience depends on, including democratic accountability and coordinated infrastructure investment [195]. A further concern is that firms operating under competitive pressure have demonstrably concealed information about the systemic vulnerabilities their activities create, prioritizing short‐term profit over transparency [196]. Additionally, even when regulation attempts to mandate resilience through minimum critical utility standards, the cost‐effectiveness for individual firms may be prohibitive when the potential harms are largely distributed across a population of end‐users. There are many protections that market mechanisms, and even regulation (without additional resourcing), cannot supply.

Another factor that becomes clear from this review is that maintaining trade and cooperation is of utmost importance, which is explicitly highlighted in many studies (e.g. [13, 54, 85]), especially for smaller countries that are not self‐sufficient in key productive sectors (e.g., food, fuel, energy, etc). Even places like Australia and New Zealand could likely struggle if they were cut off from imports of medicine, fuel and fertilizer. In the globalized world of today, no country can truly stand alone. This is also showcased by the emergence and proliferation of globally critical infrastructure like GPS, the undersea cable network or the global dependence on semiconductors from Taiwan [139] and the presence of global ‘pinch points’ where a convergence of multiple critical systems occurs [82]. These would also have to be maintained at least on some level after catastrophe hits, or all systems that rely on them would need to adapt or fail.

Besides all the factors described in this study, there are also likely others which have not been described yet. For example, Australia also has a large number of current and historic mines [197]. These could be used to avoid high UV radiation or fallout during an ASRS and give easier access to some resources after a GCIL.

A critical point for all GCRs mentioned here is also the decisions and response done directly in the aftermath of catastrophe. Preparation is important, but it will not avoid damages if immediate response is delayed or inappropriate. This means plans and negotiations have to happen before the catastrophic event and have to be stress tested and trained.

### 5.5 Interactions With Other Risks and Limitations

Considering other risks like climate change and overstepped planetary boundaries could also interact with the risks examined here [198], especially as the risks of climate change increase over time (for example in the food system [199];) and change the distribution of national resilience. For example, while Australia seems to be generally more resilient to GCRs, it is also one of the most climate vulnerable among high‐income countries due to its long coastlines, hot summers, and predilection for droughts, floods, and bushfires (it was famously described in 1908 by the poet Dorothea Mackellar as ‘a land of drought and flooding rains’) [200]. Similarly, the criteria here could come into conflict with systemic problems. For example, the EU plans to have a significant fraction of their agriculture become organic. However, organic agriculture tends to have lower yields than conventional agriculture [201], and life‐cycle analyses suggest that it generally uses more land while having similar climate impacts per unit of product on average [202]. Lower yields could result in increased starvation during a nuclear winter, as the remaining countries produce less food. Similarly, a vegetarian diet is often seen as an important contribution to stay within planetary boundaries [203]. Yet the inefficiency of meat production, the very feature that makes it environmentally costly, also means it represents a substantial latent food reserve that could, in a catastrophe, be redirected to direct human consumption.

Another important caveat is that we assess factors mostly at the national level, but resilience also varies enormously within countries. Urban areas are highly vulnerable to supply chain disruption [33, 77], while rural and remote regions within a country may be substantially more resilient. A subnational analysis could highlight other factors as more important. However, we focused on the national level, as a large part of the global population does not live in regions that could produce enough food to feed everyone [204, 205]. This means in many cases at least multi‐regional cooperation might be needed to avoid local famine. Theoretically, population relocation could also be an option, but this would likely face extreme difficulties.

Hazards also interact and the world could face several at once, or a single hazard triggering several adverse outcomes. A clear example here is a nuclear war, which can both lead to an ASRS via the soot from firestorms, but also to a GCIL via HEMP or experiencing a pandemic after a nuclear war has deteriorated the health of a large part of the global population. Another is a pandemic leading both to a GCBR and a GCIL, since people are either too ill or too scared to go to work. In these overlapping crises, it gets even harder to make a clear case for a single country being able to withstand such a catastrophe.

The results we present here are also influenced by research attention. Australia and New Zealand often come up in discussions around resilience, but many of the relevant papers are from a small group of researchers that specifically focus on these countries. This does not mean that the findings are wrong, but just that other countries might also have a relatively high resilience, but nobody has gotten around to studying them yet. Similarly, the results here could also be influenced by our expert driven literature addition by adding mainly papers for countries that the experts think are important.

The literature also shows significant coverage gaps: nuclear war and pandemics (especially COVID‐19) are studied more from a resilience perspective, while volcanic catastrophes, large‐scale cyber attacks, and HEMP are rarely discussed in the context of global risk resilience.

This review is a snapshot in time; which countries are most resilient will shift as political, economic, and environmental conditions change.

## 6 Conclusion

No single place on Earth is resilient against all global catastrophic risks, but Australia is the country most frequently identified as resilient in the literature we reviewed (Figure 8). Even this country, however, is vulnerable to specific hazards and dependent on continued international trade for fuel, medicine, and fertilizer. There is truly no place to hide.

Across the different GCR categories examined, four resilience factors appeared most consistently: geographic isolation (especially island status or the ability to enforce tight border controls), self‐sufficiency (particularly in food production), governance quality (democratic institutions, low inequality, low corruption, social cohesion), and decentralization (avoiding single points of failure in infrastructure, food systems, and decision‐making).

Of these, governance quality and decentralization stand out as both modifiable through policy and largely free of cross‐GCR trade‐offs. Investments in democratic institutions, inequality reduction, state capacity, and decentralized infrastructure improve resilience to every category of GCR examined while also making societies better places to live. The current democratic backsliding, rising inequality and increasing supply chain concentration are thus even more concerning.

Beyond strengthening these general factors, the literature identifies several concrete and currently underutilized interventions: investing in research and deployment of resilient foods, incorporating GCR into national risk assessments, risk registers and national resilience planning, establishing trade agreements that address post‐catastrophe conditions, maintaining agricultural and genetic diversity, pre‐positioning critical resources in the most resilient locations, and establishing pre‐catastrophe international cooperation frameworks.

The identification of structurally resilient states like Australia and New Zealand is no mere academic exercise. These countries could serve as anchor points for a global resilience architecture: hosting pre‐positioned food stockpiles and seed banks, maintaining industrial capacity essential for post‐catastrophe recovery, and coordinating international response. Making their role explicit, and investing accordingly, could benefit not just these countries but also increase global resilience.

The most important implication of this review remains that catastrophic events should be avoided as much as possible. Prevention is always preferable to resilience. But some GCRs, like volcanic eruptions, cannot currently be prevented. When they occur, the difference between societies that have invested in the factors identified here and those that have not will be measured in lives.

## Author Contributions

Conceptualization: F.U.J., M.R., M.B., Data curation: F.U.J., M.R., M.B., Formal analysis: F.U.J., M.R., M.B., Investigation: F.U.J., M.R., M.B., Methodology: F.U.J., M.R., M.B., Software: F.U.J., M.R., Supervision: F.U.J., Validation: F.U.J., M.R., M.B., Visualization: F.U.J., Writing – original draft: F.U.J., M.R., M.B., Writing – review & editing: F.U.J., M.R., L.K., M.C., Z.K., J.B.G.M., L.M., M.B.

## Financial Support

No financial support was received for this study.

## Conflicts of Interest

The authors declare that they have no conflicts of interest.

## Data Availability

The data that support the findings of this study are openly available in Code and Data Repository at https://zenodo.org/records/20524149, reference number https://doi.org/10.5281/zenodo.19233001.
